# Detection of Respiratory Viruses in Wastewater Samples Using Commercial Multiplex PCR Assays

**DOI:** 10.3390/v18091008

**Published:** 2026-09-13

**Authors:** Giuseppe Sberna, Maya Petricciuolo, Barbara Camilloni, Eleonora Lalle, Agnese Carnevali, Flavia Smoquina, Alessandro Graziani, Fabiano Brillo, Lavinia Fabeni, Maria Beatrice Valli, Martina Rueca, Ermanno Federici, Fabrizio Maggi, Giulia Berno

**Affiliations:** 1 Laboratory of Virology and Laboratories of Biosafety, National Institute for Infectious Diseases Lazzaro Spallanzani-IRCCS, 00149 Rome, Italygiulia.berno@inmi.it (G.B.); 2 Laboratory of Applied and Environmental Microbiology, Department of Chemistry, Biology and Biotechnology, University of Perugia, 06122 Perugia, Italyermanno.federici@unipg.it (E.F.); 3 Microbiology and Clinical Microbiology, Department of Medicine and Surgery, University of Perugia, 06122 Perugia, Italy; barbara.camilloni@unipg.it (B.C.); alessandro.graziani@collaboratori.unipg.it (A.G.)

**Keywords:** wastewater, respiratory viruses, multiplex PCR, surveillance

## Abstract

Wastewater-based epidemiology (WBE) has emerged as a supplement to clinical investigations, facilitating the acquisition of timely and non-invasive epidemiological data. In this study, a one-year WBE study was conducted in Perugia (Umbria, Italy) to evaluate respiratory viruses using multiplex assays. Fifty-two wastewater samples were collected weekly between October 2023 and September 2024 and analyzed with the Allplex™ Respiratory Panel 2/3 (for Adenovirus (AdV), Enterovirus (EV), Metapneumovirus (MPV), Parainfluenza virus types 1/2/3/4 (PIV1–4), Bocavirus (BoV), Alphacoronavirus 229E, Alphacoronavirus NL63, Betacoronavirus OC43, and Rhinovirus (RV)) and FTD respiratory pathogens 21 (only for IFV-A and RSV). The results were descriptively compared with clinical data to explore temporal concordance between wastewater and clinical surveillance. Wastewater analysis revealed frequent detection of AdV, EV, and BoV, whereas RV was detected in 67.3% of samples. AdV, EV, and BoV were detected more frequently in wastewater than in clinical samples. RSV showed a seasonal pattern in clinical samples but was only sporadically detected in wastewater, whereas PIVs were detected more frequently in clinical samples than in wastewater. By descriptive comparison, IFV-A, MPV, and non-SARS-CoV-2 coronaviruses showed peaks occurring in similar periods in wastewater and clinical samples. These results support the potential of WBE as a complementary tool to traditional clinical surveillance and suggest that commercial multiplex assays may be useful for broad, exploratory screening of respiratory viral targets in wastewater.

## 1. Introduction

Wastewater-based epidemiology (WBE) is now considered a key approach for monitoring both bacterial and viral pathogens [1]. In recent years, it has become an important tool for virus surveillance, particularly respiratory viruses, providing an early indication of virus circulation before detection in clinical specimens. Consequently, numerous countries have developed and implemented advanced molecular methods for detection of viruses in wastewater while also activating structured wastewater monitoring and surveillance systems designed to track various viral pathogens [1,2]. A clear example of this approach’s effectiveness is the recent SARS-CoV-2 pandemic, during which WBE proved valuable for tracking the virus and detecting potential new variants [3,4]. In this context, WBE has proven to be a cost-effective, non-invasive method for monitoring the spread and evolution of SARS-CoV-2, and the scientific community quickly extended the application of this approach to other seasonal respiratory viruses, such as influenza viruses (IFV), as well as other emerging pathogens [5,6]. Consequently, WBE can provide valuable insights into the control of a wider range of respiratory infections in the population [7].

In recent years, WBE has emerged as an effective supplement to conventional clinical investigations, facilitating the acquisition of timely and non-invasive epidemiological data. This approach is particularly useful as an early indicator of respiratory virus circulation, offering a temporal advantage in virus detection prior to identification in clinical specimens [3]. Supporting this, studies using real-time quantitative reverse transcription polymerase chain reaction (RT-qPCR) single-plex assays have shown that the presence of IFV RNA in wastewater follows seasonal trends consistent with those observed in clinical data, thereby underscoring the reliability of WBE monitoring as an epidemiological indicator [7].

Multiplex PCR assays, capable of simultaneous amplification of multiple genetic targets within a single reaction, could be crucial tools for the analysis of complex matrices such as urban wastewater. These tests significantly reduce analysis time, costs, and reagent usage compared to performing individual single-plex assays for each virus, all while maintaining high levels of analytical sensitivity. However, it is essential to distinguish between multiplex tests specifically developed and analytically validated for wastewater and commercial respiratory panels originally designed for clinical samples. The latter may be affected by inhibition associated with wastewater, differences in concentration and extraction efficiency, target abundance and competition among targets. Therefore, their application to wastewater should be considered exploratory in nature, unless specific analytical validation for wastewater has been performed.

A multiplex assay using droplet digital PCR (RT-ddPCR) has recently been developed and validated for the detection and quantification of IFV and human respiratory syncytial virus (RSV) in wastewater samples. This method showed excellent performance in terms of sensitivity, specificity, and resistance to inhibitors present in the environmental matrix [8]. Similarly, multiplex RT-qPCR methods, which can simultaneously detect SARS-CoV-2, IFV, and RSV in wastewater samples, have been described. These assays exhibited performance comparable to that of traditional single-plex assays, with detection limits that meet the requirements of environmental surveillance [9,10,11,12]. However, although progress has been made, using multiplex assays to monitor respiratory viruses in wastewater still presents significant technical challenges. These include the complexity of the wastewater matrix, the presence of potential PCR inhibitors, the sample variability, and the low abundance of some viruses requiring optimized extraction and amplification protocols. Additionally, international standards for data normalization are still developing, making critical evaluation of results in large-scale surveillance necessary.

Previous studies have used panels that are able to simultaneously detect a very broad range of respiratory viruses for wastewater [8,10,13,14]. The present study extends this work through a one-year, weekly WBE study conducted in the city of Perugia (Umbria, Central Italy) to evaluate the applicability and descriptive detection patterns of commercial multiplex kits (Allplex™ Respiratory Panel 2/3 and FTD respiratory pathogens 21) for respiratory virus surveillance in wastewater. Since these tests were not specifically validated for wastewater in this study, the objective was to provide a descriptive longitudinal assessment based on the frequency and temporal distribution of positive wastewater samples. These results were descriptively compared with concurrent clinical data collected in the same geographical area to explore temporal patterns across the two surveillance systems.

## 2. Materials and Methods

### 2.1. Wastewater Collection

A total of 52 wastewater samples (50 mL each) were collected weekly between October 2023 and September 2024 as part of the national SARS-CoV-2 surveillance program in wastewater (SARI project, EU Commission Recommendation 2021/472); 24 h composite samples were taken at the inlet of a wastewater treatment plant in the city of Perugia (Umbria, Italy), which has a designed capacity corresponding to a population equivalent to 90,000. Samples were stored at 4 °C and analyzed within 48 h.

### 2.2. Sample Processing and Nucleic Acid Extraction

Wastewater samples were processed according to the standardized national protocol [15] as described previously [3]. Briefly, 45 mL of heat-inactivated sample (56 °C for 30 min) was centrifuged at 4500× *g* for 30 min at 4 °C to eliminate debris. To concentrate the viral particles, 40 mL of supernatant supplemented with polyethylene glycol 8000 (8% *w*/*v*) and NaCl (0.3 M) was centrifuged at 12,000× *g* for 120 min at 4 °C. Nucleic acid extraction was carried out using the eGENE-UP system (bioMerieux, Marcy-l’Étoile, France), following the manufacturer’s instructions. To reduce the potential impact of wastewater-associated inhibitors, before molecular testing, TE-eluted nucleic acids were purified with the OneStep PCR Inhibitor Removal Kit (Zymo Research, Irvine, CA, USA). Purified extracts were supplemented with one U/μL of RiboLock RNase Inhibitor (Thermo Fisher Scientific, Waltham, MA, USA) and stored at −80 °C. The internal PCR controls included in the respective tests were interpreted according to the manufacturers’ instructions to verify the validity of the amplification and identify potential PCR inhibition during the amplification phase. These controls did not monitor viral recovery, concentration efficiency, or extraction efficiency.

### 2.3. Allplex™ Respiratory Panel 2/3

Allplex™ Respiratory Panel 2/3 (Seegene, Seoul, Republic of Korea) assays were used in this study (Table 1). Allplex is able to detect 12 respiratory targets: Adenovirus (AdV), Enterovirus (EV), Metapneumovirus (MPV), Parainfluenza virus types 1, 2, 3, and 4 (PIV1–4), Bocavirus (BoV), Alphacoronavirus 229E, Alphacoronavirus NL63, Betacoronavirus OC43, and Rhinovirus (RV). PCR analysis was carried out using the CFX96 instrument (Bio-Rad Laboratories, Hercules, CA, USA). Data analysis was conducted automatically with Seegene Viewer software (version 2.0). All procedures were carried out following the manufacturer’s recommended protocol. For graphical representation and mean calculation, an arbitrary value of 42.01 cycle threshold (Ct) was used for negative samples, as the manufacturer-specified detection limit was 42 Ct.

### 2.4. FTD Respiratory Pathogens 21

FTD respiratory pathogens 21 (FTD; Fast Track Diagnostics, Luxembourg) is a panel with five multiplex RT-PCR reactions that are able to detect 21 respiratory pathogens (Table 1). For this study only IFV-A and RSV were evaluated with this assay because this assay was available and routinely implemented in the participating laboratory for these targets during the study period. PCR analysis was carried out using the QuantStudio™ 5 Real-Time PCR System (Thermo Fisher Scientific Inc.). The remaining respiratory viral targets were analyzed using the Allplex™ Respiratory Panel 2/3. The two tests may differ in terms of target design, amplification chemistry, analytical sensitivity, and tolerance to inhibitors associated with wastewater. Therefore, the detection frequencies obtained with the two tests should not be interpreted as directly comparable measures of relative viral circulation or test sensitivity. No direct analytical comparison between the two tests was performed in wastewater. For graphical representation and/or mean calculation, an arbitrary value of 40.01 Ct was used for negative samples, as the manufacturer-specified detection limit was 40 Ct.

### 2.5. Clinical Samples

From October 2023 to September 2024, 4949 nasopharyngeal swabs (NPS) were collected from patients who attended the Santa Maria della Misericordia Hospital (Perugia, Italy). The population included patients of all age groups, both hospitalized and non-hospitalized, according to the diagnostic requests. Sample collection was performed for routine clinical diagnostic activities and was not specifically designed for the purposes of this study. Clinical samples were analyzed by extracting nucleic acids with a STARlet automated instrument (Seegene, Seoul, Republic of Korea) and then by a one-step real-time PCR using the kit Allplex™ Respiratory Panel Assays 1–3 (Seegene, Seoul, Republic of Korea) that allowed the identification of viruses such as IFV-A, RSV, AdV, EV, PIV 1–4, MPV, BoVs, RV, and non-SARS-CoV-2 coronaviruses (229E, NL63, OC43). Clinical and wastewater data were compared at the population level only. No direct linkage was established between individuals included in the clinical dataset and those contributing to the wastewater catchment. Although both datasets originated from the same geographical area (Perugia, Italy), the extent of population overlap was not quantified.

## 3. Results

Weekly wastewater samples were analyzed using multiplex PCR panels and compared with clinical NPS data collected over the same period. Overall, respiratory viruses were detected in wastewater samples (Table 2).

Across all sampling months, BoV was consistently detected in wastewater, with 100% positivity in every sample. In clinical specimens, BoV was identified at much lower frequencies, ranging from 0.0% in October and November 2023 to a peak of 1.3% in February 2024 (Supplementary Table S1). AdV showed similar persistence in wastewater, with all samples testing 100% positive except in October, November and December 2023, where positivity was slightly lower (60–75%; Supplementary Table S1). Clinical detection of AdV was more variable, with positivity rates between 0.2% and 1.8% and with a single month without detection (September 2024; Supplementary Table S1). Human RV and EV were also frequently detected in wastewater. RV was present in most months, with positivity ranging from 100% (October 2023, February, April and June 2024) to 0.0% in August 2024. Clinical RV detection was consistently higher, peaking at 24.0% in October 2023 and remaining above 3.0% in several months (Supplementary Table S1). EV was detected in all wastewater samples except for minor fluctuations, while clinical positivity remained low, generally below 1.3%, except for a peak at 3.0% in September 2024 (Supplementary Table S1). PIVs were rarely detected in wastewater, with only one positive sample in May 2024 (20%). In contrast, clinical samples showed continuous detection throughout the study period, with positivity ranging from 0.3% to 6.3%, peaking in October 2023 and April 2024 (Supplementary Table S1). RSV exhibited a clear seasonal pattern in clinical samples, with positivity starting in November 2023 (3.1%), peaking in December (10.3%), and gradually declining through April 2024. Wastewater detection of RSV was minimal, with only two positive samples recorded in January 2024 (Supplementary Table S1). Non-SARS-CoV-2 coronaviruses exhibited a peak in February 2024, both in wastewater samples (100%) and in clinical samples (5.2%; Supplementary Table S1).

**Figure 1:**
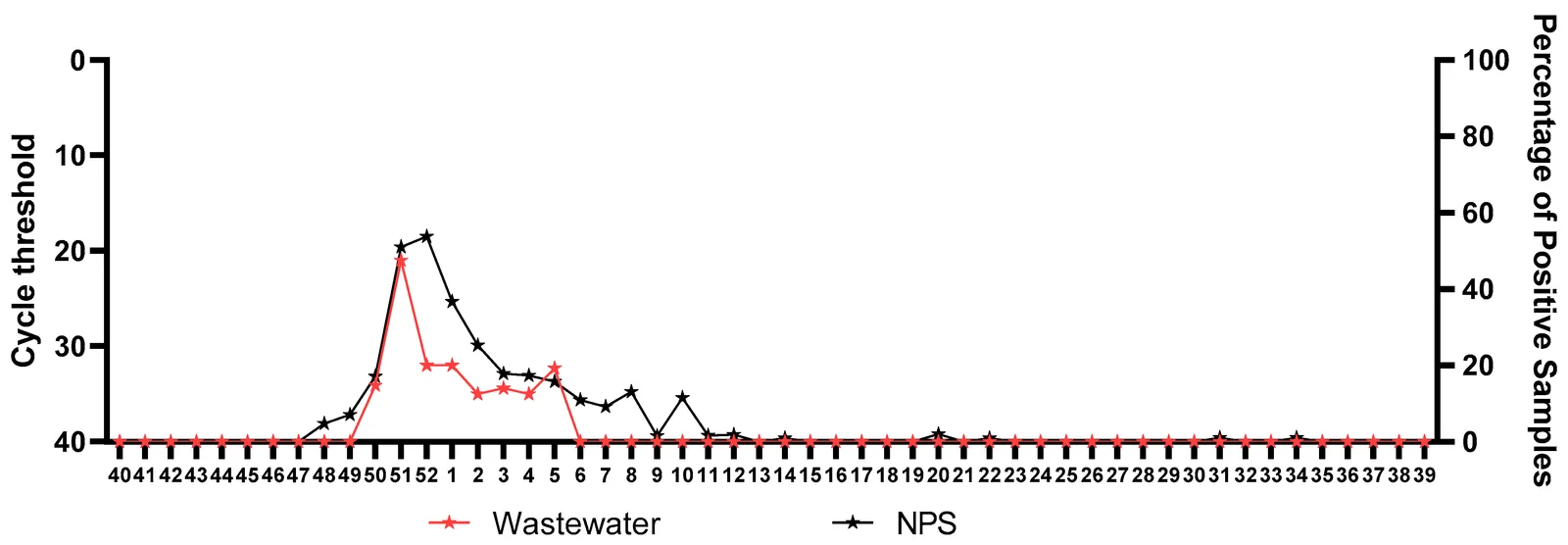
Weekly trends of IFV-A in wastewater (red line) and clinical samples (black line) from October 2023 to September 2024. IFV-A Ct-values of wastewater are plotted as weekly measurements. For graphical representation, an arbitrary value of 40.01 Ct was used for negative samples, as the manufacturer-specified detection limit was 40 Ct. This value was used solely for visualization purposes and does not represent a measured Ct-value or a viral concentration. The dual-axis representation is provided for descriptive visualization of temporal trends only and does not imply a quantitative relationship between wastewater Ct-values and clinical positivity rates.

Furthermore, descriptive visualization of wastewater Ct-values and clinical positivity rates showed that IFV-A, identified using the FTD panel, was detected in wastewater between week 49 of 2023 and week 6 of 2024, coinciding with the peak of clinical positivity observed during the same period. After February 2024, IFV-A detection declined sharply in both matrices (Figure 1).

**Figure 2:**
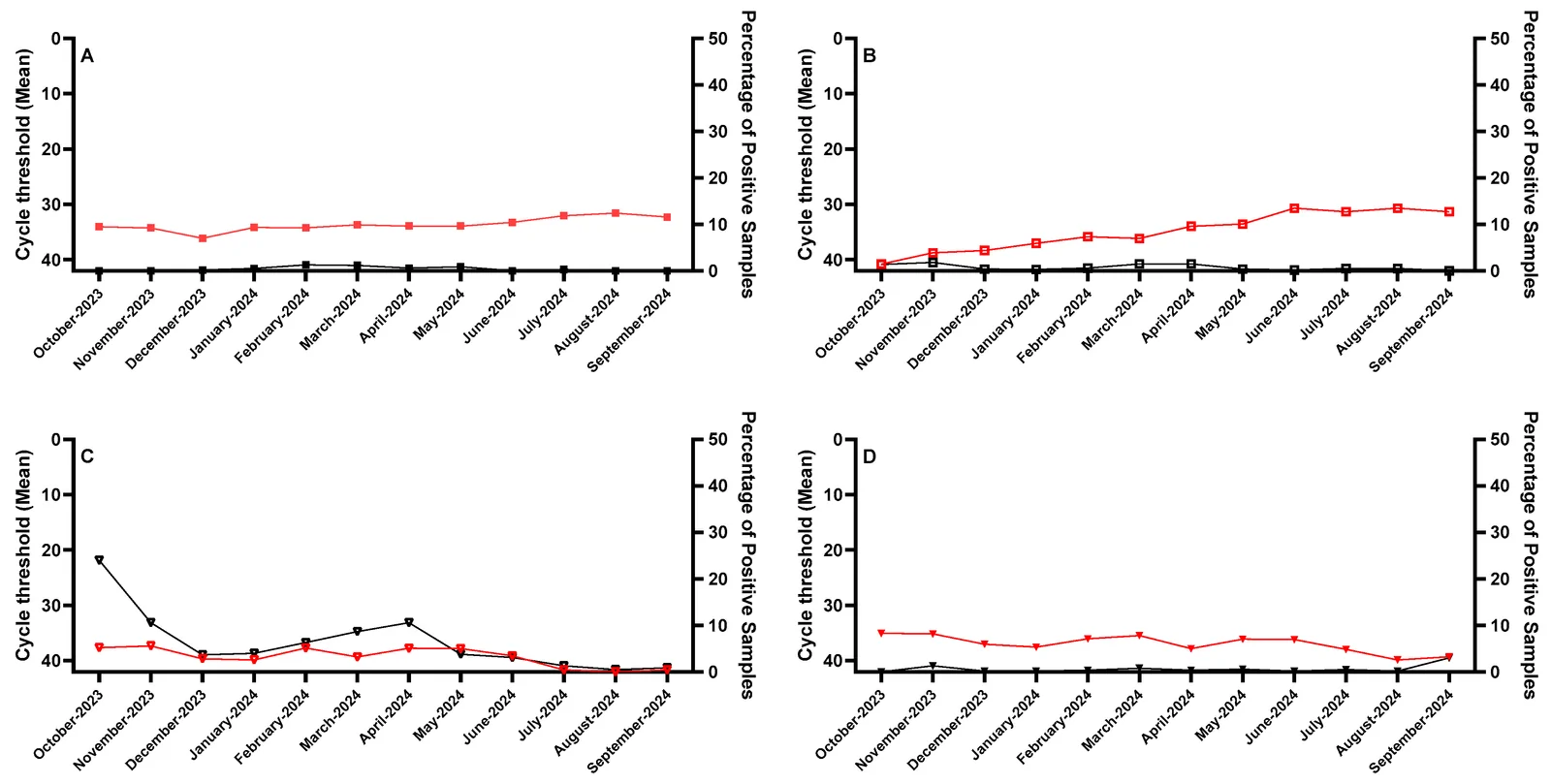

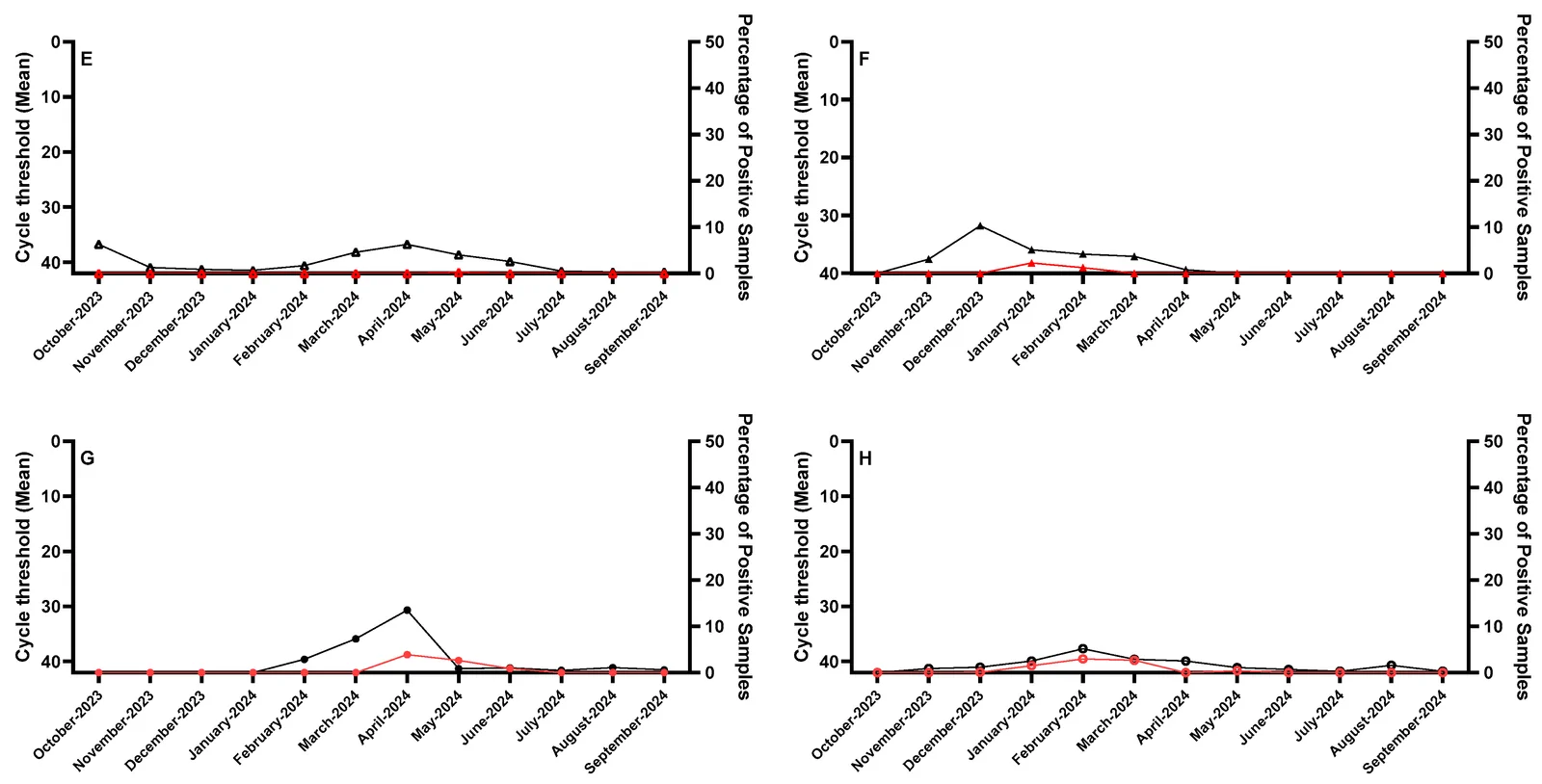
Trends of BoV (**A**), AdV (**B**), RV (**C**), EV (**D**), PIV (**E**), RSV (**F**), MPV (**G**), and non-SARS-CoV-2 coronaviruses (**H**) in both wastewater (red line) and clinical samples (black line). Data reported for non-SARS-CoV-2 coronaviruses and PIV refer exclusively to Alphacoronavirus 229E and PIV-3, respectively, since they were the only non-SARS-CoV-2 coronavirus and PIVs detected in the analyzed samples. Ct-value for wastewater represents the mean Ct-values obtained from the weekly samples collected during the corresponding calendar month. For graphical representation and mean calculation, an arbitrary value of 42.01 Ct was used for negative samples, as the manufacturer-specified detection limit was 42 Ct. For RSV graphical representation and mean calculation, an arbitrary value of 40.01 Ct was used for negative samples, as the manufacturer-specified detection limit was 40 Ct. These values were used solely for visualization purposes and do not represent measured Ct-values or viral concentrations. The dual-axis representation is provided for descriptive visualization of temporal trends only and does not imply a quantitative relationship between wastewater Ct-values and clinical positivity rates.

BoV, AdV, and EV showed a nearly constant trend in wastewater levels despite an almost complete absence in the clinical samples (Figure 2A,B,D), while RV displayed a similar trend between the two matrices (Figure 2C). Conversely, PIVs and RSV are more prevalent in clinical samples than in wastewater (Figure 2E,F). MPV showed similar temporal trends in wastewater and clinical samples, with both showing increased detection during spring 2024, peaking in April (Figure 2G); the same pattern can be observed for non-SARS-CoV-2 coronaviruses, with both wastewater and clinical samples peaking in February 2024 (Figure 2H). Overall, the dataset reveals continuous detection of BoV and AdV in wastewater, frequent RV and EV presence, sporadic detection of PIVs and RSV in wastewater, and evident seasonal peaks for MPV, non-SARS-CoV-2 coronaviruses, and IFV-A in both wastewater and clinical samples.

## 4. Discussion

The integration between environmental and clinical surveillance is essential, and WBE offers an additional view to conventional case-based systems. Numerous studies have demonstrated strong correlations between viral RNA levels in wastewater and clinical surveillance trends, particularly for SARS-CoV-2, confirming the usefulness of WBE as both a complementary epidemiological tool and, in some contexts, a potential early warning system [4,10]. The public health value of environmental surveillance has also been confirmed by recent European findings. In 2024, wastewater surveillance networks operating in Finland, Spain, Poland, Germany, and the United Kingdom detected the presence of type 2 vaccine-derived poliovirus (cVDPV2) in the absence of reported clinical cases [16,17], and, in 2025, the same network also identified wild poliovirus type 1 (WPV1) in a wastewater sample from Hamburg, despite Europe being considered polio-free [18]. These episodes clearly highlight the role of wastewater as a sentinel system for the early identification of emerging or re-emerging health threats.

Multiplex systems are attractive because they allow simultaneous screening for multiple pathogens and can broaden the scope of surveillance. The present study therefore aimed to evaluate the applicability and descriptive detection patterns of commercial multiplex PCR assays, originally designed for clinical respiratory samples (e.g., NPS), for the surveillance of respiratory viruses in urban wastewater, comparing environmental evidence with contemporaneous clinical data from the same geographical area. This approach provides additional information on the practical applicability, limitations, and pathogen-dependent interpretation of these panels in wastewater surveillance.

Across the study period, AdV, EV, and BoV were detected in a high percentage of wastewater samples, often exceeding their detection rates in clinical samples, while RV was detected in 67.3% of the samples. This trend should not be interpreted as a direct estimate of respiratory circulation, since signals in wastewater incorporate the pathogen-specific shedding, route of excretion, persistence, solid–liquid distribution, and analytical recovery [19,20,21]. For example, AdV typing is particularly important; in fact, enteric AdV-F40/41 may account for a substantial fraction of the adenoviruses detected in wastewater, while respiratory adenovirus types may exhibit distinct patterns correlated with epidemic outbreaks [22,23]. Since the Allplex™ Respiratory Panel 2 does not distinguish between adenovirus types, it is not possible to determine the relative contributions of respiratory versus enteric adenoviruses [24]. EVs can be shed in feces and urine and can be detected in wastewater throughout the year [25,26]. Therefore, the frequent detection of EVs observed in this study may reflect enteric shedding and prolonged excretion at the community level rather than a simple respiratory infection. Furthermore, it cannot be ruled out that asymptomatic or minimally symptomatic individuals who do not seek medical care may also contribute to the discrepancy between wastewater detection and clinical detection. BoV has been detected in both respiratory and fecal samples, and prolonged or recurrent shedding following primary infection has been reported [27,28]. Therefore, the frequent detection of BoV in wastewater may reflect a combination of respiratory and enteric transmission, including mild or asymptomatic infections [29].

Environmental persistence may also play a role, as non-enveloped viruses such as AdV, EV, and BoV can persist longer than many enveloped viruses under certain environmental conditions [30]. However, detection in wastewater is also influenced by solid–liquid partitioning, concentration, and extraction efficiency, PCR inhibition, and the sensitivity of the target-specific test [21,31]. Therefore, the observed pattern may reflect a combination of biological and methodological factors.

In contrast, viruses associated with more symptomatic acute respiratory illness [32] showed a more evident alignment between wastewater and clinical data. IFV-A, MPV and non-SARS-CoV-2 coronaviruses showed peaks occurring in broadly similar periods in wastewater and clinical samples, suggesting that wastewater detection patterns may broadly reflect temporal patterns of community circulation of the pathogens. These results are consistent with previous studies demonstrating a good correlation between environmental and clinical data for IFV-A [7,9]. In this context, data from the Italian RespiVirNet network for the 2023–2024 season indicate that the circulation of IFV-A and main respiratory viruses in Italy and Umbria peaked between December 2023 and February 2024, with a predominance of IFV-A and significant MPV circulation [33]. Seasonal and regional variations must be distinguished from the performance of analytical tests. Changes in local viral circulation, transmission patterns, and the catchment area population can influence the detection rates observed in wastewater, the distributions of Ct-values, and comparisons with clinical data. Studies conducted in different geographic settings have reported distinct seasonal patterns and differences in the timing and magnitude of respiratory virus signals, particularly for influenza, RSV, PIVs, seasonal coronaviruses, and adenoviruses [9,22,29,34]. Therefore, results obtained from a single wastewater treatment plant and during a single year of sampling should not be directly generalized to other regions or seasons.

Regarding RSV and PIVs, these viruses were consistently detected in clinical samples but only sporadically in wastewater. These data differ from some published studies, which found PIVs in wastewater to correlate with clinical data [9,34] and RSV detected in wastewater even during mild or asymptomatic infections not identified by healthcare systems [8]. However, several factors likely explain this discrepancy. Both viruses are enveloped and can therefore be relatively fragile and may show lower environmental persistence under some wastewater conditions [30]. Methodological aspects may also play a role, as the heat inactivation step and the removal of solids may potentially disrupt viral envelopes and/or eliminate virions adsorbed to particulates; however, their individual effects were not assessed in the present study. Accordingly, candidate pretreatment workflows for enveloped viruses should be compared experimentally, including rapid low-temperature processing with and without heat inactivation, retention or separate processing of solids, and the inclusion of an exogenous enveloped virus recovery control. Again, shedding dynamics could have an impact: gastrointestinal shedding of RSV and PIVs is limited or intermittent, resulting in wastewater concentrations near the detection limit and reducing the likelihood of consistent positivity even when clinical incidence is high.

In the literature, the results on the ability of wastewater to anticipate virus positivity compared to clinical data are conflicting: some studies report a clear time lag compared to clinical cases [5,35], while others show a close overlap between wastewater and clinical data trends [36]. In the present study, the trends of wastewater and clinical data were substantially overlapping, with no clear temporal advance. This observation could be attributed to several factors. First, the lower analytical sensitivity of the multi-target assays, optimized for respiratory swabs, may not be sufficient to consistently detect certain low abundance targets at the very start of community seeding [12]. Despite all these limitations, adopting multiplexed approaches for wastewater analysis represents a significant evolution from the traditional single-plex approach, allowing broader respiratory pathogen screening from the same extract with reduced turnaround time, costs, and reagent consumption [11]. At the same time, the environmental application of clinical PCR panels remains technically challenging due to matrix complexity, inhibitor profiles, and the absence of universally accepted normalization protocols. Further work will be essential to optimize concentration/extraction for enveloped targets, to validate assay performance specifically on wastewater, and to harmonize reporting units and controls [8,10].

This study has some limitations. First, the commercial multiplex assays were originally developed for clinical specimens and were not subjected here to wastewater-specific analytical validation. Wastewater-specific limits of detection, viral recovery efficiency, concentration efficiency, nucleic acid extraction efficiency, and matrix effects were therefore not determined. No exogenous process-recovery or separate extraction control was included; the assay-specific PCR internal controls monitored only amplification validity and potential PCR inhibition. Therefore, recovery, concentration, nucleic acid extraction efficiency, and matrix effects were not quantified, and wastewater Ct-values were interpreted descriptively rather than as recovery-corrected quantitative measurements. These limitations reflect the recognized methodological challenges in wastewater monitoring, as recovery efficiency, PCR inhibition, and analytical sensitivity can vary depending on concentration, the extraction workflow, and the target virus [20,21,37,38]. Second, no formal correlation or time lag analysis was performed between wastewater Ct-values and clinical positivity rates; thus, comparisons between the two datasets should be considered descriptive. Third, IFV-A/RSV and the remaining respiratory viruses were evaluated using different commercial assays. Differences in target design, amplification chemistry, analytical sensitivity, and potential tolerance to wastewater-associated inhibitors may have influenced detection frequencies. Finally, pathogen-specific differences in shedding route, duration of excretion, solid–liquid partitioning, and environmental stability may strongly influence wastewater detection [19,30,31].

In addition to improving and validating PCR-based workflows, future wastewater surveillance may benefit from emerging rapid and decentralized sensing technologies. Photoelectrochemical and microfluidic CRISPR-based platforms could potentially complement laboratory-based PCR by enabling faster, multiplex, and on-site detection. An automated robot-driven photoelectrochemical platform has been used to detect SARS-CoV-2 Omicron BA.5 in sewage, whereas a LOC-CRISPR microfluidic system has demonstrated multiplex detection of respiratory viruses and variants in clinical or controlled samples rather than wastewater [39,40]. Tellurium nanosheets have shown stable photoresponse properties that may be relevant to the development of photoactive sensing materials, although this materials study did not evaluate viral detection or wastewater [41]. However, these technologies remain prospective and require wastewater-specific validation of matrix tolerance, recovery, sensitivity, specificity, reproducibility, and throughput before routine implementation.

Overall, these results support the potential of WBE as a complementary tool to traditional clinical surveillance and provide useful insights into the interpretation of environmental sample results based on the biological and epidemiological characteristics of different respiratory viruses. Combining environmental and clinical surveillance may provide complementary information for epidemiological interpretation: environmental data can guide health authorities toward areas or periods where clinical investigations should be intensified, while clinical data may provide context for interpreting environmental detection patterns. However, this study did not demonstrate a lead-time advantage of wastewater surveillance over clinical surveillance. Commercial multiplex respiratory PCR assays may be useful for broad, exploratory screening of multiple viral targets in wastewater; however, their interpretation requires caution due to the absence of wastewater-specific analytical validation, assay-dependent sensitivity and pathogen-specific differences in shedding and environmental stability. Further multicenter studies conducted in different geographic regions and during different respiratory virus seasons, along with validation protocols specific to wastewater, are needed to assess the reproducibility and generalizability of these detection patterns and to define the role of commercial multiplex panels in routine wastewater surveillance.

## Figures and Tables

**Table 1:** Viral targets and differentiation capabilities of the commercial respiratory panels used in this study.

Assay	Viral Targets Covered	Strain Differentiation Capability	Use in The Present Study
Allplex™ Respiratory Panel 2	AdV, EV, MPV, and PIVs	PIVs are reported as separate serotypes: PIV1, PIV2, PIV3 and PIV4. AdV, EV, and MPV are reported without serotype or strain differentiation	All listed targets were evaluated
Allplex™ Respiratory Panel 3	BoV, non-SARS-CoV-2 coronaviruses and RV	Non-SARS-CoV-2 coronaviruses are reported separately as Alphacoronavirus 229E, Alphacoronavirus NL63, Betacoronavirus OC43. BoV and RV are reported as grouped targets, without genotype or strain differentiation	All listed targets were evaluated
FTD respiratory pathogens 21	IFV-A, IFV-B, RV, PIVs, MPV, BoV, RSV, AdV, EV, and parechovirus	The panel differentiates between IFV-A, IFV-A H1N1 swl, IFV-B. PIVs are reported as separate serotypes: PIV1, PIV2, PIV3 and PIV4. Non-SARS-CoV-2 coronaviruses are reported separately as Alphacoronavirus 229E, Alphacoronavirus NL63, Betacoronavirus OC43, Betacoronavirus HKU1. RSV A/B and MPV A/B targets are included. No further strain or genotype differentiation was performed	Only IFV-A virus and RSV were evaluated

**Table 2:** Detection of respiratory viruses in wastewater samples.

	BoV	AdV	RV	EV	PIVs	RSV	MPV	Non-SARS-CoV-2 Coronaviruses	IFV-A
N. Positive/N. Total	52/52	48/52	35/52	47/52	1/52	2/52	8/52	12/52	8/52
Percentage of positive samples	100.0	92.3	67.3	90.4	1.9	3.8	15.4	23.1	15.4

## Data Availability

The original contributions presented in the study are included in the article; further inquiries can be directed to the corresponding author.

## Supplementary Materials

The following supporting information can be downloaded at https://www.mdpi.com/article/10.3390/v18091008/s1, Table S1: Comparison of viral detection in wastewater and clinical samples.
