# Supplementary material for: Detection of Respiratory Viruses in Wastewater Samples Using Commercial Multiplex PCR Assays
Source: Viruses. 2026 Sep 13;18(9):1008. doi: 10.3390/v18091008 (PMC13611849; doi:10.3390/v18091008)
Supplement: Supplementary file 1 [file viruses-18-01008-s001.zip › viruses-4514449-supplementary.pdf]

Supplementary Table S1. Comparison of viral detection in wastewater and clinical samples.

Months	Epidemiological week (from—to)	BoV		AdV		MPV	
		WW: N. Positive/N. Total (%)	NPS: N. Positive/N. Total (%)	WW: N. Positive/N. Total (%)	NPS: N. Positive/N. Total (%)	WW: N. Positive/N. Total (%)	NPS: N. Positive/N. Total (%)
October 2023	40–44	5/5 (100.0)	0/221 (0.0)	3/5 (60.0)	3/221 (1.4)	0/5 (0.0)	0/221 (0.0)
November 2023	45–48	4/4 (100.0)	0/226 (0.0)	3/4 (75.0)	4/226 (1.8)	0/4 (0.0)	0/226 (0.0)
December 2023	49–52	4/4 (100.0)	1/427 (0.2)	3/4 (75.0)	2/427 (0.4)	0/4 (0.0)	0/427 (0.0)
January 2024	01–05	5/5 (100.0)	3/605 (0.5)	5/5 (100.0)	2/605 (0.3)	0/5 (0.0)	0/605 (0.0)
February 2024	06–09	4/4 (100.0)	6/478 (1.3)	4/4 (100.0)	3/478 (0.6)	0/4 (0.0)	14/478 (2.9)
March 2024	10–13	4/4 (100.0)	6/520 (1.2)	4/4 (100.0)	8/520 (1.5)	1/4 (25.0)	38/520 (7.3)
April 2024	14-17	4/4 (100.0)	3/481 (0.6)	4/4 (100.0)	7/481 (1.5)	3/4 (75.0)	65/481 (13.5)
May 2024	18–22	5/5 (100.0)	4/468 (0.9)	5/5 (100.0)	2/468 (0.4)	3/5 (60.0)	4/468 (0.9)
June 2024	23–26	4/4 (100.0)	0/419 (0.0)	4/4 (100.0)	1/419 (0.2)	1/4 (25.0)	4/419 (1.0)
July 2024	27–31	5/5 (100.0)	1/396 (0.3)	5/5 (100.0)	2/396 (0.5)	0/5 (0.0)	2/396 (0.5)
August 2024	32–35	4/4 (100.0)	0/376 (0.0)	4/4 (100.0)	2/376 (0.5)	0/4 (0.0)	4/376 (1.1)
September 2024	36–39	4/4 (100.0)	0/332 (0.0)	4/4 (100.0)	0/332 (0.0)	0/4 (0.0)	2/332 (0.6)

Months	Epidemiological week (from—to)	RV		EV		non-SARS-CoV-2 coronaviruses	
		WW: N. Positive/N. Total (%)	NPS: N. Positive/N. Total (%)	WW: N. Positive/N. Total (%)	NPS: N. Positive/N. Total (%)	WW: N. Positive/N. Total (%)	NPS: N. Positive/N. Total (%)
October 2023	40–44	5/5 (100.0)	53/221 (24.0)	5/5 (100.0)	0/221 (0.0)	0/5 (0.0)	0/221 (0.0)
November 2023	45–48	3/4 (75.0)	24/226 (10.6)	4/4 (100.0)	3/226 (1.3)	0/4 (0.0)	2/226 (0.9)
December 2023	49–52	3/4 (75.0)	16/427 (3.7)	4/4 (100.0)	1/427 (0.2)	1/4 (25.0)	5/427 (1.2)
January 2024	01–05	4/5 (80.0)	24/605 (4.0)	4/5 (80.0)	1/605 (0.2)	3/5 (60.0)	15/605 (2.5)
February 2024	06–09	4/4 (100.0)	30/478 (6.3)	4/4 (100.0)	2/478 (0.4)	4/4 (100.0)	25/478 (5.2)
March 2024	10–13	2/4 (50.0)	45/520 (8.7)	4/4 (100.0)	4/520 (0.8)	3/4 (75.0)	15/520 (2.9)
April 2024	14-17	4/4 (100.0)	51/481 (10.6)	3/4 (75.0)	2/481 (0.4)	0/4 (0.0)	12/481 (2.5)
May 2024	18–22	4/5 (80.0)	18/468 (3.8)	5/5 (100.0)	3/468 (0.6)	1/5 (20.0)	5/468 (1.1)
June 2024	23–26	4/4 (100.0)	13/419 (3.1)	4/4 (100.0)	1/419 (0.2)	0/4 (0.0)	3/419 (0.7)
July 2024	27–31	1/5 (20.0)	5/396 (1.3)	4/5 (80.0)	2/396 (0.5)	0/5 (0.0)	1/396 (0.3)
August 2024	32–35	0/4 (0.0)	2/376 (0.5)	3/4 (75.0)	2/376 (0.5)	0/4 (0.0)	6/376 (1.6)
September 2024	36–39	1/4 (25.0)	3/332 (0.9)	3/4 (75.0)	10/332 (3.0)	0/4 (0.0)	1/332 (0.3)

Months	Epidemiological week (from—to)	PIV		RSV		IFV-A	
		WW: N. Positive/N. Total (%)	NPS: N. Positive/N. Total (%)	WW: N. Positive/N. Total (%)	NPS: N. Positive/N. Total (%)	WW: N. Positive/N. Total (%)	NPS: N. Positive/N. Total (%)
October 2023	40–44	0/5 (0.0)	14/221 (6.3)	0/5 (0.0)	0/221 (0.0)	0/5 (0.0)	0/221 (0.0)
November 2023	45–48	0/4 (0.0)	3/226 (1.3)	0/4 (0.0)	7/226 (3.1)	0/4 (0.0)	2/226 (0.9)
December 2023	49–52	0/4 (0.0)	4/427 (0.9)	0/4 (0.0)	44/427 (10.3)	3/4 (50.0)	153/427 (35.8)
January 2024	01–05	0/5 (0.0)	4/605 (0.7)	2/5 (40.0)	31/605 (5.1)	5/5 (100.0)	145/605 (24.0)
February 2024	06–09	0/4 (0.0)	8/478 (1.7)	0/4 (0.0)	20/478 (4.2)	0/4 (0.0)	70/478 (14.6)
March 2024	10–13	0/4 (0.0)	24/520 (4.6)	0/4 (0.0)	19/520 (3.7)	0/4 (0.0)	63/520 (12.1)
April 2024	14-17	0/4 (0.0)	30/481 (6.3)	0/4 (0.0)	4/481 (0.8)	0/4 (0.0)	14/481 (2.9)

May 2024	18–22	1/5 (20.0)	19/468 (4.0)	0/5 (0.0)	0/468 (0.0)	0/5 (0.0)	5/468 (1.1)
June 2024	23–26	0/4 (0.0)	11/419 (2.6)	0/4 (0.0)	0/419 (0.0)	0/4 (0.0)	0/419 (0.0)
July 2024	27–31	0/5 (0.0)	2/396 (0.5)	0/5 (0.0)	0/396 (0.0)	0/5 (0.0)	0/396 (0.0)
August 2024	32–35	0/4 (0.0)	1/376 (0.3)	0/4 (0.0)	0/376 (0.0)	0/4 (0.0)	2/376 (0.5)
September 2024	36–39	0/4 (0.0)	1/332 (0.3)	0/4 (0.0)	0/332 (0.0)	0/4 (0.0)	0/332 (0.0)

WW: wastewater; NPS: nasopharyngeal swab; AdV: Adenovirus; EV: Enterovirus; MPV: Metapneumovirus; PIV: Parainfluenza virus; BoV: Bocavirus (BoV); non-SARS-CoV-2 coronaviruses: Alphacoronavirus 229E, Alphacorona-virus NL63, Betacoronavirus OC43; RV: Rhinovirus; RSV: Respiratory syncytial virus; IFV-A: Influenza virus A.
